# Hyponatremia and its associated factors in children admitted to the pediatric intensive care unit in eastern Ethiopia: a cross-sectional study

**DOI:** 10.1186/s12887-023-04118-7

**Published:** 2023-06-20

**Authors:** Yeshi Berhanu, Turina Yusuf, Ahmed Mohammed, Fentahun Meseret, Betelhem Demeke Habteyohans, Ayichew Alemu, Gadissa Tolosa, Mulualem Keneni, Fitsum Weldegebreal, Assefa Desalew

**Affiliations:** 1 Department of Pediatrics and Child Health, School of Medicine, College of Health and Medical Sciences, Haramaya University, Harar, Ethiopia; 2 Department of Pediatrics and Child Health Nursing, School of Nursing and Midwifery, College of Health and Medical Sciences, Haramaya University, Harar, Ethiopia; 3 School of Medical Laboratory Sciences, College of Health and Medical Sciences, Haramaya University, Harar, Ethiopia

**Keywords:** Hyponatremia, Hiwot Fana Comprehensive Specialized University Hospital, Children, Ethiopia

## Abstract

**Background:**

Hyponatremia is a serious problem that leads to substantial increases morbidity and mortality in critically ill children. The identification of risk factors, implementation of preventive measures, and timely diagnosis and management are crucial to reduce adverse events related to hyponatremia. Despite the higher burden of the problem in Ethiopia, evidence related to the risk factors for hyponatremia among children in Ethiopia is limited; in particular, no study has been identified in eastern Ethiopia. Therefore, we aimed to determine the magnitude of hyponatremia and its associated factors in children admitted to the pediatric intensive care unit at the Hiwot Fana Comprehensive Specialized University Hospital.

**Methods:**

A facility-based cross-sectional study was conducted using 422 medical records of pediatric patients admitted to the pediatric intensive care unit at Hiwot Fana Comprehensive Specialized University Hospital from January 2019 to December 2022. Medical records were reviewed to collect data. Data were analyzed using a statistical package for social sciences (SPSS) version 26. A binary logistic regression model with an adjusted odds ratio (aOR) and a 95% confidence interval (CI) was used to identify factors associated with the outcome variable. Statistical significance was set at p < 0.05.

**Results:**

The magnitude of hyponatremia was 39.1% (95% CL: 34.4–43.8%). The age of the child (aOR = 2.37;95% CL:1.31–4.31), diagnosis of sepsis (aOR = 2.33; 95% CL:1.41–3.84),   surgical procedures (aOR = 2.39; 95% CL:1.26–4.56), nutritional status (aOR = 2.60; 95% CL:1.51–4.49), and length of hospital stay (aOR = 3.04; 95% CL: 1.73–5.33) were factors significantly associated with hyponatremia.

**Conclusions:**

Four out of ten children admitted to pediatric intensive care units had hyponatremia. Hyponatremia was significantly associated with the age of the child, malnutrition, sepsis, surgical procedures, and length of hospital stay. To reduce the burden of hyponatremia and associated mortality, attention should be focused on improving the care of malnourished children, and those with sepsis, and the quality of postoperative monitoring services. Moreover, intervention strategies aimed at reducing the burden of hyponatremia should target the identified factors.

## Background

Hyponatremia is defined as a plasma sodium level of less than 135 mmol/L. It is the most common electrolyte disorder encountered in clinical practice and is associated with significant morbidity and mortality [1–3]. Hyponatremia can cause neurological dysfunction, decreased mental function, cerebral edema, osteoporosis, fractures, gait disturbances, and falls [4–6]. Hyponatremia affects up to 22% of hospitalized patients and up to 67.2% of hospitalized in pediatric intensive care units ( PICUs) [7]. Hyponatremia accounts for 32.5% of mortality after admission to the PICUs. However, early diagnosis and treatment of hyponatremia can reduce morbidity and hospitalization times [6, 8]. Underlying medical conditions increase the risk of moderate to severe hyponatremia and death [3, 9–11].

Hyponatremia is associated with overall poor outcomes in pediatric populations [11]. The problems associated with delays in the provision of appropriate care can result in seizures, acute psychosis, permanent brain damage, and brainstem herniation, leading to coma and death [3, 6, 12, 13]. Appropriate treatment of pediatric hyponatremia necessitates knowledge of the etiology of hyponatremia; the child’s effective circulating volume, hemodynamic stability, the severity of symptoms, and the duration and rate of sodium concentration change [13]. Low sodium levels should be treated based on the underlying cause, and treatment with hypertonic sodium, fluid restriction, and treatment of the underlying disease leads to complete recovery [14, 15].

Hyponatremia has multifactorial causes and can be associated with salt and water loss [1, 15, 16]. Free water consumption, and underlying conditions that cause no osmotic stimulation for the synthesis of vasopressin [16]. Children are especially susceptible to clinical hyponatremia and hyponatremia encephalopathy and have a poor prognosis if treatment is not started on time [17, 18]. Critically ill patients admitted to the PICU with underlying medical conditions have abnormal sodium and potassium levels [19].

The most frequent electrolyte abnormalities are hyponatremia associated with central nervous system disorders (52.9%), gastrointestinal disorders (17.6%), and sepsis (11.8%) [7, 20]. Moreover, malaria, gastroenteritis, pneumonia, malnutrition, neonatal sepsis [21], postsurgical status, prolonged PICU stay, fluid intake, and mechanical ventilation were other significantly associated factors [22, 23]. Hyponatremia can increase the length of hospital stay by 1–2 days and correlates with an increased risk of intensive care unit admission [22, 24].

Preventive measures for hyponatremia include checking plasma sodium levels 1–2 weeks after starting thiazide, selective serotonin reuptake inhibitor (SSRI), and selective norepinephrine reuptake inhibitor (SNRI) therapy, especially in patients at high risk for hyponatremia; avoiding hypotonic fluids and thiazides in people with high fluid or low protein intake and measuring plasma sodium levels in all hospitalized patients on admission [25, 26]. Clinicians should monitor daily plasma sodium levels in patients with hyponatremia or in those at high risk [25]. Furthermore, the American Academy of Pediatrics recommends the use of isotonic fluids for the maintenance of intravenous fluids in patients aged 28 days to 18 years to reduce the risk of developing hyponatremia, which can occur with hypotonic fluids [27].

Few studies had investigated the burden of hyponatremia in low and middle-income countries, particularly Ethiopia where many pediatric patients are admitted to the PICUs. The lack of sodium level studies in these settings may lead to inappropriate treatments, especially with supportive fluid therapy, which should consider electrolyte concentrations [14, 28]. Early recognition of hyponatremia and its associated factors may be enhanced by supportive therapy to improve outcomes. Studies investigating the magnitude and associated factors in children admitted to the PICUs are warranted. Therefore, this study aimed to assess the magnitude of hyponatremia and address the associated factors among patients admitted to the PICU at Hiwot Fana Comprehensive Specialized University Hospital.

## Methods

### Study setting, design, and population

This retrospective cross-sectional study was conducted at Hiwot Fana Comprehensive Specialized University Hospital in eastern Ethiopia. This is a teaching referral hospital at Haramaya University, located 526 km from Addis Ababa. This facility provides health services for an estimated six million people in eastern Ethiopia. The hospital was established during the Italian invasion and became a teaching and referral hospital at Haramaya University in 2014. There are different wards and clinics within the hospital, including the pediatric ward, PICU, neonatal intensive care unit (NICU), internal medicine, surgery, gynecology, and obstetrics. This study was conducted among children admitted to the PICU between January 2019 and December 30, 2022. All medical records of children admitted to the PICU between January 2019 and December 2022 were the source population. However, the medical records of children with pre existing electrolyte disturbances, such as chronic kidney disease, and medical records without electrolyte determination were excluded from this study.

### Sample size determination and sampling procedure

The sample size was computed using a single proportion formula by considering 50% hyponatremia as a proportion (P) because no study was previously conducted in Ethiopia. We considered the following assumptions: 95% confidence interval (CI) and marginal error of 5%. The calculated sample size was 384; we added a 10% nonresponse rate, and the final sample size was 422. A simple random sampling technique was used to select participants. A total of 628 patients were admitted to the PICU of the hospital from January 2019 to December 2022. Of these, 422 were included in the study using computer-generated simple random sampling techniques and fulfilled the inclusion criteria.

### Data collection

Data were collected by two BSc nurses and supervised by two senior MSc nurses. Data were collected from medical chart review using a validated data abstraction checklist adapted from previous studies [10, 21, 22]. The tools contain information on the sociodemographic factors of the child such as; age, sex, and place of residence. Serum electrolyte profile; sodium. Admission diagnosis; central nervous system disorders, cardiovascular disorders, gastrointestinal disorders, endocrine disorders, respiratory disorders, postsurgical cases, renal disorders, sepsis, severe acute malnutrition, poisoning, and hematologic disorders. Hospital interventions; surgery, hyponatremia fluid therapy, mechanical ventilation, length of hospital stay, and outcomes. Serum electrolyte levels were measured following the standard operating procedure. Serum Sodium value: Normal Range: 135 to 145 mmol/L, Hyponatremia: <135 mmol/L Hypernatremia: >145 mmol/L [17]. Weight for length/height, between 3SD and 2SD – normal and below 2SD- malnourished; BMI for age, between 3SD and 2SD – normal and below 2SD- malnourished.

### Data quality assurance

To ensure the quality of data, a pretest was conducted with 5% of the sample size at Dil Chora Hospital’s pediatric intensive care unit. Two days of training were provided to all data collectors and supervisors. The data collection process was closely supervised. Supervisors and principal investigators checked the completeness of each questionnaire daily. During data cleaning, a logical checking technique was employed to identify errors. Finally, double data entry was performed to verify data consistency.

### Data processing and statistical analysis

The collected data were entered into Epi Data 4.6 and analyzed using the Statistical Package for Social Sciences (SPSS) version 26. Frequency, mean and proportion were used for the descriptive analysis. A binary logistic regression model was used to determine the factors associated with the outcome variable. All variables with p ≤ 0.25 in the bivariable logistic regression were entered into the final multivariable analysis to control confounders. The goodness of fit of the model was tested using the Hosmer‒Lemeshow test (> 0.05). A multicollinearity test was performed to determine the correlation between the independent variables using variance inflation factors (VIF > 10). The adjusted odds ratio (aOR) with 95% confidence intervals (CI) and a p-value of less than 0.05 were considered statistically significant.

## Results

### Sociodemographic characteristics

The medical records of 422 children were reviewed. The age of the study subjects ranged from 29 days to 15 years and the mean and standard deviation (SD) age was 3 ± 3.7 years. Of these 422 participants, 187 (44.3%) were aged less than 5 years. Among the study participants, 243 (57.6%) were male while the remaining 179 (42.4%) were female. Nearly two thirds of the study participants, 251(59.5%) were from urban areas [Table 1].

**Table 1:** Sociodemographic and nutritional characteristics of pediatric patients admitted to the PICU in Hiwot Fana Comprehensive Specialized University Hospital, Ethiopia, 2022 (n = 422)

Variable	category	Frequency	Percent
Sex	Male	245	58.0
	Female	177	41.9
Age	Less than 1 year	143	33.9
	1–5 yrs	175	41.5
	5–15 yrs	104	24.6
Place of residence	Urban	251	59.5
	Rural	171	40.5
MUAC	Severely malnourished	127	30.1
	Moderately malnourished	108	25.6
	Well-nourished	112	26.5
	Not assessed	75	17.8
WFH/L	Severely malnourished	134	31.8
	Moderately malnourished	149	35.3
	Well-nourished	139	32.9
BMI for age	Severely malnourished	52	12.3
	Moderately malnourished	37	8.8
	Well-nourished	15	3.6
	Not assessed	318	75.4

## Magnitude of hyponatremia

**Fig. 1:**
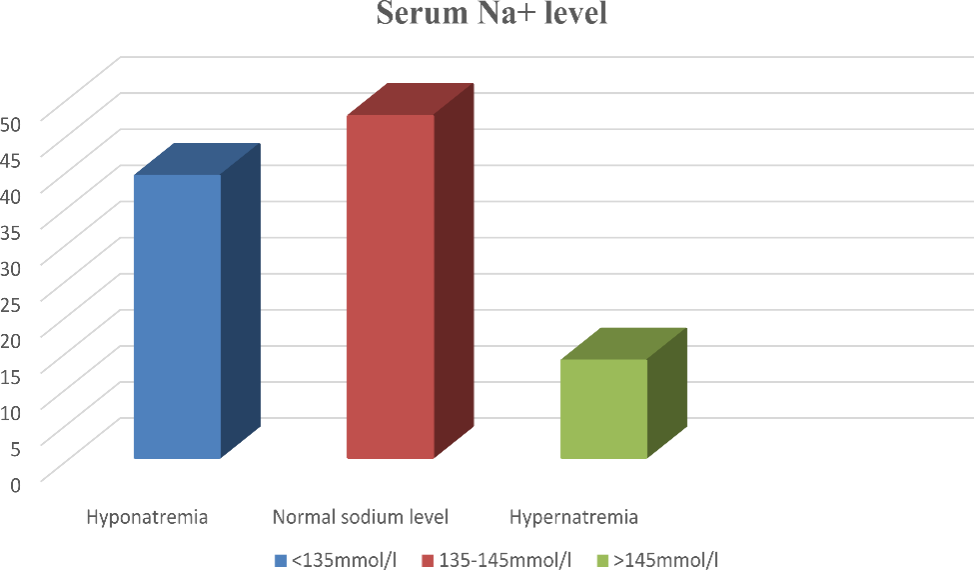
Serum sodium level of pediatric patients admitted to the PICU in Hiwot Fana Comprehensive Specialized Hospital, eastern Ethiopia, 2022 (n = 422) List of tables

Of the 422 medical records reviewed, 165 (39.1%) had hyponatremia with 95% CL: 34.4–43.8%). The mean sodium concentration was 136 mmol/l. Two hundred (47.4%) patients had a normal range of sodium concentration [Fig. 1].

**Table 2:** Comorbidities status of pediatric patients admitted to the PICU in Hiwot Fana Comprehensive Specialized University Hospital, Ethiopia, 2022, (n = 422)

		Frequency	Percent
Respiratory	yes	28	6.6
	No	394	93.4
Respiratory types	Bronchiolitis	7	25.0
	Para pneumonic effusion	9	32.1
	Empyema	1	3.6
	Acute severe asthma	11	39.3
GI	Yes	45	10.7
	No	377	89.3
GI types	Shock	9	20.0
	Acute liver failure	6	13.3
	Hepatic encephalopathy	1	2.2
	Chronic liver disease	4	8.9
	Others	25	55.6
Renal	Yes	33	7.8
	No	389	92.1
Renal types	AKI	21	63.6
	Acute glomerulonephritis	4	12.1
	Nephrotic syndrome	5	15.2
	Congenital abnormalities	2	6.1
	Uremic encephalopathy	1	3.0
Hemato-oncology	Yes	6	1.4
	No	416	98.6
Hemato-oncology types	Malignancy Hemolytic anemia	4 2	66.7 33.3

**Table 3:** Common interventions for children admitted to the PICU in Hiwot Fana Comprehensive Specialized University Hospital, Ethiopia, 2022, (n = 422)

		Frequency	Percent
Hospital stay	< 1 week	118	28.0
	1–2 weeks	154	36.5
	> 2 weeks	150	35.5
Surgery	Yes	69	16.4
	No	353	83.6
Surgery types	Laparotomy	22	31.9
	Colostomy	5	7.2
	VP shunt	6	8.7
	Others	36	52.1
Fluid	Yes	110	26.1
	No	312	73.9
Fluid types	Normal saline	42	38.1
	NS and D5 W	29	26.4
	R/L	3	2.7
	Maintenance fluid	36	32.7
Mechanical ventilator	Yes No	7 415	1.7 98.3
Outcome	Discharged improved	214	50.7
	Discharged against	104	24.6
	Referred	11	2.6
	Death	93	22.0

**Table 4:** Factors associated with hyponatremia among children admitted to the PICU in Hiwot Fana Comprehensive Specialized Hospital, Ethiopia, 2022, (n = 422)

Variables	Hyponatremia		cOR(95%CI)	aOR (95%CI)	p-value
	Yes	No			
Age of the patients					
Less than 1 year	67	76	1.81 (1.01–3.06)	1.86 (1.00-3.46)	0.05
1–5 yrs	87	88	2.03 (1.22–3.37)	2.37 (1.31–4.31)**	0.004
5–15 yrs	34	70	1	1	
Sex					
Male	114	131	2.03 (1.22–3.37)	1.07 (0.69–1.66)	0.75
Female	74	103	1	1	
MUAC					
Severely malnourished	73	54	2.96 (1.74-5.00).	2.60 (1.51–4.49)**	0.001
Moderately malnourished	49	59	1.69 (0.97–2.95)	1.8 (1.04–3.14)**	0.036
Well-nourished	35	77	1	1	
Renal disorders					
Yes	19	14	1.76 (0.86–3.62)	1.02 (0.44–2.37)	0.95
No	169	220	1	1	
Sepsis					
Yes	70	49	2.24 (1.45–3.45)	2.33 (1.41–3.84)**	0.001
No	118	185	1	1	
Duration of hospital stay					
< 1 week	37	81	1	1	
1–2 weeks	57	97	1.28 (0.77–2.13)	1.12 (0.64–1.95)	0.68
> 2 weeks	94	56	3.67 (2.2–6.12)	3.04 (1.73–5.33)**	0.00
Surgical procedure					
Yes	44	25	2.55 (1.49–4.36)	2.39 (1.26–4.56)**	0.008
No	144	209	1	1	
GIT disorders					
Yes	22	23	1.21 (0.65–2.25)	1.27 (0.59–2.72)	0.53
No	166	211	1	1	
CVS disorders					
Yes	16	28	0.68 (0.35–1.3)	1.19 (0.54–2.6)	0.65
No	172	206	1	1	
Place of residence					
Urban	121	130	1.44 (0.97–2.14)	1.48 (0.95–2.3)	0.78
Rural	67	104	1	1	

cOR:- crude odds ratio, aOR:- adjusted odds ratio, MUAC:- middle upper arm circumference **=p value < 0.05

### Common morbidities at admission

Nearly half of the patients, 206 (48.8%) showed altered levels of consciousness. More than one-third of the patients, 176 (41.7%) had central nervous system disorders at admission, with meningitis accounting for 146 (82.9%) of the cases. More than one-fifth of the study participants 97 (22.9%) were diagnosed with sepsis at admission and 29 (29.9%) had severe sepsis. Only 44 (10.4%) participants had cardiovascular disorders, and heart failure accounted for 36 (81.8%). One-third of the patients, 119 (28.2%) had gastrointestinal related problems. Only 3 (0.7%) had endocrine disorders of which 2 (66.7%) had diabetic ketoacidosis (DKA) and the remaining 1(33.3%) had hypoglycemia. Four (0.9%) had poisoning   [Table 2].

### Hospital interventions 

Regarding hospital stay, the patients stayed in the hospital for an average of 12 days. One-third 154 (36.5%) of the children stayed for 1 to 2 weeks. Of  422 patients, 69 (16.4%) underwent surgery, and 22 (31.9%) underwent laparotomy [Table 3].

### Factors associated with hyponatremia

Multivariable logistic regression analysis showed that; the age of the child, Mid-Upper Arm Circumference (MUAC), sepsis, surgery, and duration of hospital stay were significantly associated with hyponatremia. Children aged less than 5 years were 2.37 times (aOR = 2.37; 95% CI; 1.31–4.31) more likely to develop hyponatremia than children aged 5 and 15 years. Severely malnourished children who were measured by MUAC were 2.60 times (aOR = 2.6; 95% CI: 1.51–4.49) more likely to be hyponatremic than well-nourished children. Moreover, pediatric patients who were diagnosed with sepsis were 2.33 times (aOR = 2.32; 95% CI: 1.41–3.83) more likely to suffer from hyponatremia than their counterparts. Furthermore, children who underwent surgical procedures and were admitted to the PICU were 2.39 times (aOR = 2.39; 95% CI: 1.26–4.56) more likely to develop hyponatremia than those who did not undergo surgical procedures. In addition, patients who stayed in the PICU for more than 2 weeks were 3.04 times (aOR = 3.04; CI 1.73–5.33) more likely to develop hyponatremia than those who stayed for less than a week [Table 4].

## Discussion

Hyponatremia in critically ill children leads to a substantial increase in morbidity and mortality [1–3]. Evidence suggested that a delay in recognition and treatment of hyponatremia can lead to poor prognosis [17, 18]. Identification of risk factors, implementation of preventive measures, and timely diagnosis with management are crucial to minimize adverse events related to hyponatremia. This study aimed to assess the magnitude of hyponatremia and its associated factors among children admitted to the PICU at the Hiwot Fana Comprehensive Specialized University Hospital. The magnitude of hyponatremia was 39.1% (95% CI: 34.4–43.8%). In multivariable analysis, age less than five years, MUAC, diagnosis of sepsis, undergoing surgical procedures, and length of hospital stay were identified as independently associated with hyponatremia.

The findings of this study are consistent with those studies conducted in Egypt (36%) [29], and Nigeria (39.6%) [30]. However, this study is inconsistent with a study conducted in Addis Ababa, Ethiopia (16.7%) [22]. This variation could be due to the difference in the comorbidities that the child was admitted with, and the method of serum sodium measurement. Most laboratories use a direct measurement of sodium. This could also be hyponatremia in critically ill children, which may reflect an endogenous state of sodium dysregulation, iatrogenic causes, or both [19].

Furthermore, the present findings indicate that critically ill children aged less than 5 years were two times more likely to have hyponatremia than those aged 5–15 years. This finding is supported by studies conducted in Taiwan [31], and Nigeria [30]. This could be because younger children are at high risk for the development of hyponatremia due to their small body size, hence their high water concentration, lower glomerular filtration rate, reduced proximal tubular reabsorption of sodium, and increased arginine vasopressin levels in response to illness [32].

In addition, malnourished children are three times more likely to develop hyponatremia than their counterparts. This finding is in line with studies conducted in India [33], Bangladesh [34], and Kenya [21]. This may be due to severely malnourished children; most children have excess total body sodium despite low serum sodium levels thus, masking sodium overload [35].

Moreover, this study found that a length of hospital stay of more than two weeks was three times increase the risk of hyponatremia. This finding is in agreement with studies conducted in Saudi Arabia [29, 36]. This might be explained by the likelihood of acquiring hospital-acquired infections and receiving a large amount of fluid during the long hospital stay. Furthermore, the more patients stayed in the hospital, the more likely they were to be complicated and prone to take different drugs that can even cause hyponatremia [37].

Furthermore, children diagnosed with sepsis were twice as at risk of hyponatremia. These findings are consistent with those of a study conducted in India [2]. This could be due to sepsis resulting in dilution of the extracellular space with retained exogenous fluid secondary to disrupted cellular membrane integrity as well as the development of renal insufficiency which may lead to hyponatremia [38].

In addition, patients who underwent surgery were twice at risk of developing hyponatremia. This is comparable with studies conducted in the United States of America [36], Croatia [39], and India [40]. This may be because surgical patients receive a large amount of fluid both pre and postoperatively. Furthermore, postoperative hyponatremia is provoked by surgical stress, which causes a syndrome of inappropriate antidiuretic hormone levels in almost everyone, often promoting water retention for several days [41].

## Conclusions 

Four out of ten children admitted to pediatric intensive care unit had hyponatremia. Hyponatremia was found to be significantly associated with the age of child, malnutrition, sepsis, surgical procedures, and longer length of hospital stay in the pediatric intensive care unit.

To reduce the burden of hyponatremia and associated mortality, attention should be directed toward improving the care of malnourished children, and those with sepsis, and the quality of postoperative monitoring services.

In numerous occasions, hyponatremia is found incidentally when serum electrolytes are obtained during clinical evaluation for severly ill patients. So early diagnosis of more risk patients for the occurrence of hyponatremia and early intervention is very important.

Moreover, intervention strategies aimed at reducing the burden of hyponatremia should target the identified factors.

## Data Availability

Data will be available upon request from the corresponding author.

## List of Abbreviations

ADH: Anti-diuretic hormone
aOR: Adjusted odds ratio
COR: Crude odds ratio
DKA: Diabetic ketoacidosis
MUAC: Mid-upper arm circumference
PICU: Pediatric intensive care unit
SIADH: Syndrome of inappropriate antidiuretic hormone
IHREC: Institutional Health Research Ethical Committee
SPSS: Statistical Package for Social Sciences.
